# Neutrophil-to-Lymphocyte Ratio as a Potential Biomarker to Managing Type 2 Diabetes Mellitus and Predicting Disease Progression

**DOI:** 10.7759/cureus.55227

**Published:** 2024-02-29

**Authors:** Abdul Muqeeth Mohammed, Mohammed Khaleel, Padmaja R. M., Qader A Jalily, Kalyani Dhanekula, Mummareddi Dinesh Eshwar

**Affiliations:** 1 Clinical Biochemistry, Mahavir Institute of Medical Sciences, Vikarabad, IND; 2 Microbiology, Mahavir Institute of Medical Sciences, Vikarabad, IND

**Keywords:** serum biomarkers, neutrophil to lymphocyte ratio (nlr), serum ferritin, glycated hemoglobin (hba1c), type1 diabetes mellitus

## Abstract

Introduction

Diabetes is a chronic disease that causes dysregulation of blood glucose. Type 2 diabetes mellitus (T2DM) could result in long-term inflammatory conditions that affect different organs of the body. Despite the availability of diagnostic markers like glycated hemoglobin (HbA1c) for T2DM, it is essential to find an appropriate marker that could predict long-term complications. This study evaluates the potential role of neutrophil-to-lymphocyte ratio (NLR) in predicting disease progression and treatment responses.

Methods

This case-control study was carried out among 160 T2DM patients and 132 non-diabetic persons. Blood samples were collected from each participant and were processed for hemoglobin, HbA1c, iron, ferritin, and complete blood picture (NLR).

Results

The study showed that there was a significant variation in the serum levels of ferritin (264.8±611.6 ng/ml versus 168.3±364.7 ng/ml, p=0.392), iron (4.095±8.851 mcg/dl versus 55.20±37.62 mcg/dl, p=0.0111), and HbA1c (8.169±1.635% versus 5.668±0.5260% p<0.0001) among T2DM patients compared to non-diabetic persons. The NLR values (4.189±4.154 versus 4.095±8.851, p=0.009) among patients with T2DM significantly varied with that of non-diabetic persons. A significant negative correlation was noticed between the serum levels of iron and NLR (r=-0.17, p=0.014) and a positive correlation was noticed between HbA1c and NLR (r=0.19, p=0.014). The serum levels of iron revealed a significant positive correlation with the serum levels of ferritin (r=0.24, p=0.002) and hemoglobin percentage (r=0.41, p=0.008). HbA1c revealed a significant positive correlation with NLR (r=0.19, p=0.014). Additionally, a significant negative correlation was observed between iron with NLR (r=-0.17, p=0.029) and hemoglobin percentage with NLR (r=-0.30, p=0.005). However, no such correlation was demonstrated among non-diabetic persons. With an accuracy of 89.85% and high sensitivity and specificity, NLR showed diagnostic accuracy like HbA1c.

Conclusions

NLR demonstrated equivalent efficacy to HbA1c in predicting glycemic control. Since diabetes affects different organs of the body, evaluating NLR probably predicts inflammation. Therefore, NLR could be useful in the management of T2DM and in predicting long-term complications.

## Introduction

Diabetes is a chronic illness characterized by elevated blood glucose/sugar levels. Uncontrolled diabetes over a long period could affect different organs of the body like the heart, eyes, and kidneys. Diabetes also disturbs the metabolism of a person and disrupts the nervous system. According to the World Health Organization (WHO), more than 420 million people could be living with diabetes. Recently, there has been an increasing prevalence of diabetes throughout the world, especially among people living in low- and middle-economy countries like India [1]. 

There are two types of diabetes; diabetes mellitus (DM) and diabetes insipidus. DM is the most predominant type of diabetes which is further classified into type 1 diabetes mellitus (T1DM), type 2 diabetes mellitus (T2DM), and gestational diabetes among other types. T2DM is the most common type of diabetes which is generally an age-related disease that affects older people. The several causes for the development of T2DM include genetic factors (chromosome 2, 7, 12, 13, 17, mitochondrial DNA, etc.), inflammatory conditions (pancreatitis, cystic fibrosis, etc.), infectious causes (congenital rubella, cytomegalovirus, etc.), hormonal disorders (hyperthyroidism, phaeochromocytoma, etc.), drug-related factors (glucocorticoids, thiazides, etc.), and immunity-related (anti-insulin receptor antibodies, etc.) [2]. Other modifiable factors like physical activity, stress, sleep, and food may also contribute to the development of T2DM [3]. 

Research in the past found evidence of disturbed iron metabolism among T2DM patients. It was identified that serum activities of ferritin significantly (p=0.0003) varied between people without T2DM (40.853±15.55 ng/mL), and people with controlled (73.3±56.6 ng/ml) and uncontrolled T2DM (269.8±347.1 ng/ml) [4]. There is some evidence that T2DM could cause chronic low-grade inflammation that may involve the development of long-term complications attributed to T2DM. The neutrophil-to-lymphocyte ratio (NLR) was recently suggested as a potential marker that predicts inflammation among T2DM patients. A study from Turkey that assessed NLR among T2DM patients (median: 2.44) and compared with controls (median: 1.5) demonstrated a significant difference (p <0.001) [5]. 

Glycated hemoglobin (HbA1c) is an established biomarker to diagnose and manage T2DM. A recent study reaffirmed the diagnostic efficacy of HbA1c activities where a higher than 6.5% value of HbA1c could be an indicator for diagnosing T2DM and predicting T2DM-related complications like nephropathy (kidney disease) and retinopathy (eye disease) [6]. 

Despite the availability of biomarkers in diagnosing and predicting the disease progression among T2DM patients, the role of NLR in the diagnosis and management of T2DM gained significance in the recent past. Therefore, this study was carried out to evaluate NLR, serum ferritin, iron, HbA1c, and hemoglobin activities in T2DM patients and compare them with a control group without T2DM. Additionally, this study assessed the correlation between the parameters tested and their statistical significance along with the diagnostic efficacy of NLR compared with HbA1c.

## Materials and methods

A prospective case-control study was conducted in the Department of Biochemistry, Mahavir Institute of Medical Sciences, Vikarabad, Telangana, India, from December 2022 to May 2023. The ethical issues involved in this study were reviewed and approved by the Institutional Ethics Committee, Mahavir Institute of Medical Sciences (approval number: MIMS/IEC/2023/115).

The study included 292 subjects (132 non-diabetic persons and 160 T2DM patients) attending the Department of Endocrinology and Department of Internal Medicine, Mahavir Institute of Medical Sciences General Hospital, Vikarabad. All the subjects included in the study belonged to the age group of 25-75 years, were of either sex, and were selected by simple random sampling method. All patients with abnormal lipid profiles secondary to hypothyroidism, alcoholic liver disease, renal failure, nephrotic syndrome, and patients on drugs like glucocorticoids, estrogens, and progesterone, and patients with a history of familial dyslipidemia, pregnant women and smokers were excluded from the study. Informed oral consent was taken from all individuals.

Diabetes was diagnosed based on the current American Diabetes Association (ADA) diagnostic criteria for diabetes, which suggests HbA1c levels of 6.5% or more as diabetes. A venous blood sample was collected from all study participants. In this study, 5 mL of blood (3 mL drawn into a plain vacutainer-red cap and 2 mL drawn into an ethylenediamine tetraacetic acid (EDTA) vacutainer-lavender cap) was collected from each participant for a complete blood picture and estimation of HbA1C, serum ferritin, serum iron. HbA1c was measured by high-performance liquid chromatography method using a D-10 analyzer (Bio-Rad Laboratories, Inc., Hercules, California, United States). Hemoglobin estimation was by spectrophotometry and neutrophil and lymphocyte count was carried out by the DxH 800 autoanalyzer (Beckman Coulter, Inc., Brea, California, United States), NLR was calculated and serum ferritin estimation was performed by chemiluminescence method using the DxI 600 analyzer (Beckman Coulter, Inc.), serum Iron was estimated by 2,4,6-tripyridyl-S-triazine (TPTZ) using the DxC 700 AU analyzer (Beckman Coulter, Inc.).

Statistical analysis

The data collected were entered into a Microsoft Office 2019 Excel sheet (Microsoft Corporation, Redmond, Washington, United States). Data were analyzed using IBM SPSS Statistics for Windows, Version 23.0 (Released 2015; IBM Corp., Armonk, New York, United States). The data were presented as mean ± standard deviation. A student’s unpaired t-test was applied for the comparison of parameter means between T2DM patients and non-diabetic persons. Pearson’s coefficient of correlation was calculated to determine the correlation between all variables in each group. Receiver operating characteristic (ROC) curve and area under the curve (AUC) were used to test the diagnostic performance of NLR against HbA1c in each group. A p-value less than 0.05 was considered significant.

## Results

The mean age of male T2DM patients was 65.80 ± 10.74 years, while that of female T2DM patients was 59.55 ± 13.28 years and that of male non-diabetic individuals was 53.03 ± 15.82 years. The mean values of HbA1C in the non-diabetic male population (5.692 ± 0.5186 %) and diabetic males (8.178 ± 1.499 %) were found to be statistically significant (p < 0.05). Moreover, there was a significant variation (p < 0.05) in the Iron levels between T2DM patients (47.12 ± 33.23 mcg/dl) and non-diabetic controls (62.67 ± 35.82 mcg/dL). The detailed depiction of HbA1c, hemoglobin level (Hb%), ferritin, iron, and NLR among T2DM patients and non-diabetic participants is shown in Table 1. 

**Table 1:** Age and gender demographic data Mann-Whitney unpaired t test, *Statistically significant (p<0.05) T2DM: type 2 diabetes mellitus; NLR: neutrophil-to-lymphocyte ratio; HbA1C: glycated hemoglobin; Hb%: hemoglobin level

	Males			Females		
Parameters	T2DM patients (n=89), mean ± SD	Non-diabetic controls (n=60), mean ± SD	p-value	T2DM patients (n=71) , mean ± SD	Non-diabetic controls (n=72), mean ± SD	p-value
Age (years)	65.80 ± 10.74	61.50 ± 14.44	0.1218	59.55 ± 13.28	53.03 ± 15.82	0.0159*
HbA1C (%)	8.178 ± 1.499	5.692 ± 0.5186	<0.0001*	8.158 ± 1.801	5.649 ± 0.5350	<0.0001*
Hb%	10.52 ± 2.144	11.04 ± 2.460	0.2094	10.13 ± 1.823	10.65 ± 1.896	0.1001
Ferritin (ng/ml)	293.8 ± 667.4	253.0 ± 502.6	0.5452	228.5 ± 536.0	97.70 ± 156.2	0.1125
Iron (mcg/dl)	47.12 ± 33.23	62.67 ± 35.82	0.0046*	47.93 ± 44.05	48.98 ± 38.20	0.2587
NLR	3.910 ± 3.502	5.311 ± 12.45	0.6125	4.539 ± 4.852	3.082 ± 3.657	0.0020*

The study showed that there was a significant variation in the serum levels of ferritin (264.8±611.6 ng/ml versus 168.3±364.7 ng/ml, p=0.392), iron (4.095±8.851 mcg/dl versus 55.20±37.62 mcg/dl, p=0.0111), and HbA1c (8.169±1.635% versus 5.668±0.5260% p<0.0001) among T2DM patients compared to non-diabetic persons. The NLR values (4.189±4.154 versus 4.095±8.851, p=0.009) among patients with T2DM significantly varied with non-diabetic persons, as shown in Table 2.

**Table 2:** Comparison of parameters among diabetic and non-diabetic subjects. Mann Whitney test, * Statistically significant (p<0.05) T2DM: type 2 diabetes mellitus; NLR: neutrophil-to-lymphocyte ratio; HbA1c: glycated hemoglobin; Hb%: hemoglobin level

Parameters	T2DM patients (n=160), Mean ± SD	Non-diabetic controls (n=132), Mean ± SD	p-value
Ferritin (ng/ml)	264.8 ± 611.6	168.3 ± 364.7	0.3925
Iron (mcg/dl)	47.48 ± 38.28	55.20 ± 37.62	0.0111*
HbA1c (%)	8.169 ± 1.635	5.668 ± 0.5260	<0.0001*
Hb%	10.35 ± 2.011	10.83 ± 2.171	0.0563
NLR	4.189 ± 4.154	4.095 ± 8.851	0.0097*

A significant negative correlation was noticed between the serum levels of iron and NLR (r=-0.17, p=0.014) and a positive correlation was noticed between HbA1c and NLR (r=0.19, p=0.014). The serum levels of iron revealed a significant positive correlation with the serum levels of ferritin (r=0.24, p=0.002) and Hb% (r=0.41, p=0.008). HbA1c revealed a significant positive correlation with NLR (r=0.19, p=0.014). Additionally, a significant negative correlation was observed between iron and NLR (r=-0.17, p=0.029) and Hb% and NLR (r=-0.30, p=0.005). The parameters were tested and their correlation with NLR are given below in Table 3.

**Table 3:** Parameters tested and their co-relation with NLR among the diabetic subjects (N=160) r=Pearsons correlation, *Statistically significant (p<0.05) NLR: neutrophil-to-lymphocyte ratio; HbA1c: glycated hemoglobin; Hb%: hemoglobin level

Parameter	Statistical Inference	Ferritin	Iron	HbA1c	Hb%	NLR
Ferritin	r value	1	0.24	0.09	0.14	0.10
	p-value		0.002	0.234	0.074	0.212
Iron	r value	0.24	1	-0.06	0.41	-0.17
	p-value	0.002		0.475	9.704e-008	0.029
HbA1c	r value	0.09	-0.06	1	-0.23	0.19
	p-value	0.234	0.475		0.003	0.014
Hb%	r value	0.14	0.41	-0.23	1	-0.30
	p-value	0.074	9.704e-008	0.003		9.527e-005
NLR	r value	0.10	-0.17	0.19	-0.30	1
	p-value	0.212	0.029	0.014	9.527e-005	

However, no such correlation was demonstrated among non-diabetic persons. The serum activities of iron positively correlated with ferritin (r=0.323, p=0.0002) and Hb% (r=0.291, p=0.001). Additionally, there was a significant negative correlation of Hb% with NLR (r=-0.230, p=0.008) and a significant positive correlation of Hb% with serum iron (r=0.291, p=0.0007) among non-diabetic participants. The details involving the inter-correlation between the parameters tested among non-diabetic persons are detailed in Table 4.

**Table 4:** Parameters tested and their co-relation among the non-diabetic subjects (N=132). r=Pearsons correlation, * Statistically significant (p<0.05) NLR: neutrophil-to-lymphocyte ratio; HbA1c: glycated hemoglobin; Hb%: hemoglobin level

Parameter	Statistical Inference	Ferritin	Iron	HbA1c	Hb%	NLR
Ferritin	r value	1	0.323	0.018	-0.113	0.035
	p-value		0.0002	0.835	0.199	0.692
Iron	r value	0.323	1	-0.042	0.291	-0.010
	p-value	0.0002		0.634	0.001	0.912
HbA1c	r value	0.018	-0.042	1	0.052	0.018
	p-value	0.8354	0.6339		0.551	0.836
Hb%	r value	-0.113	0.291	0.052	1	-0.230
	p-value	0.1986	0.0007	0.551		0.008
NLR	r value	0.035	-0.010	0.018	-0.230	1
	p-value	0.6916	0.9123	0.836	0.008	

**Figure 1:**
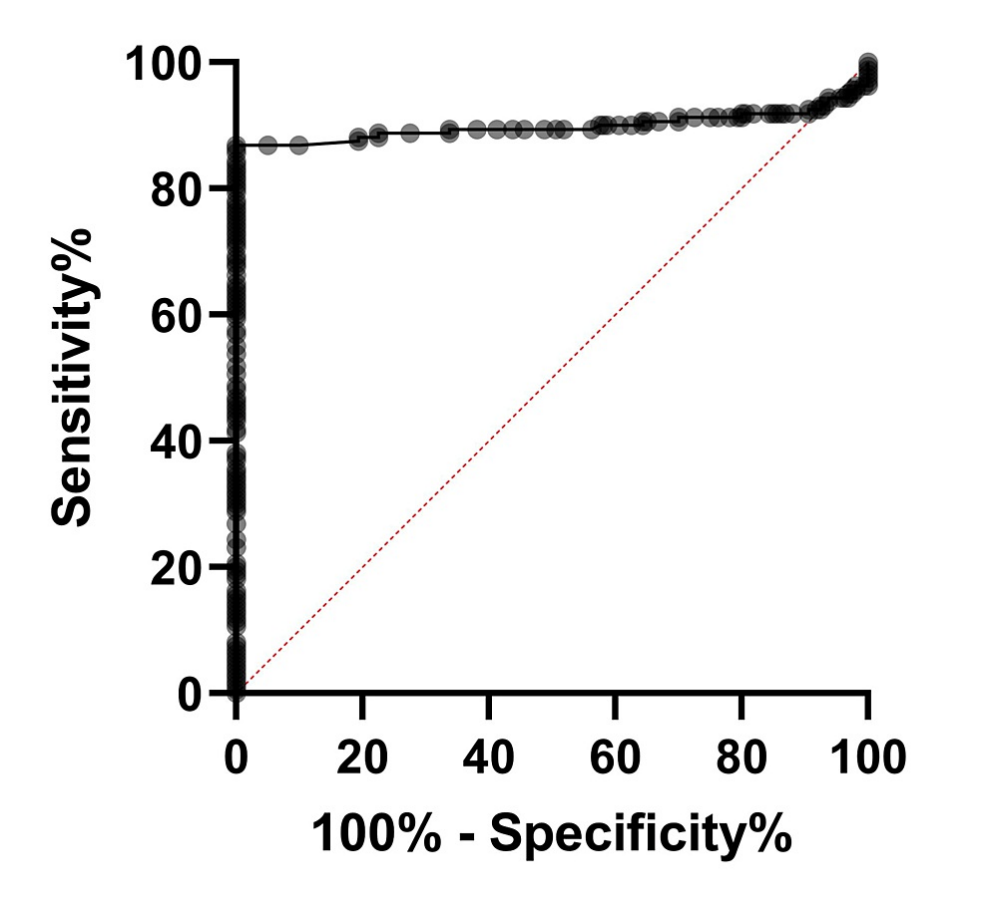
ROC curve of NLR vs HbA1C in T2DM patients ROC: receiver operating characteristic; T2DM: type 2 diabetes mellitus; NLR: neutrophil-to-lymphocyte ratio; HbA1c: glycated hemoglobin

**Figure 2:**
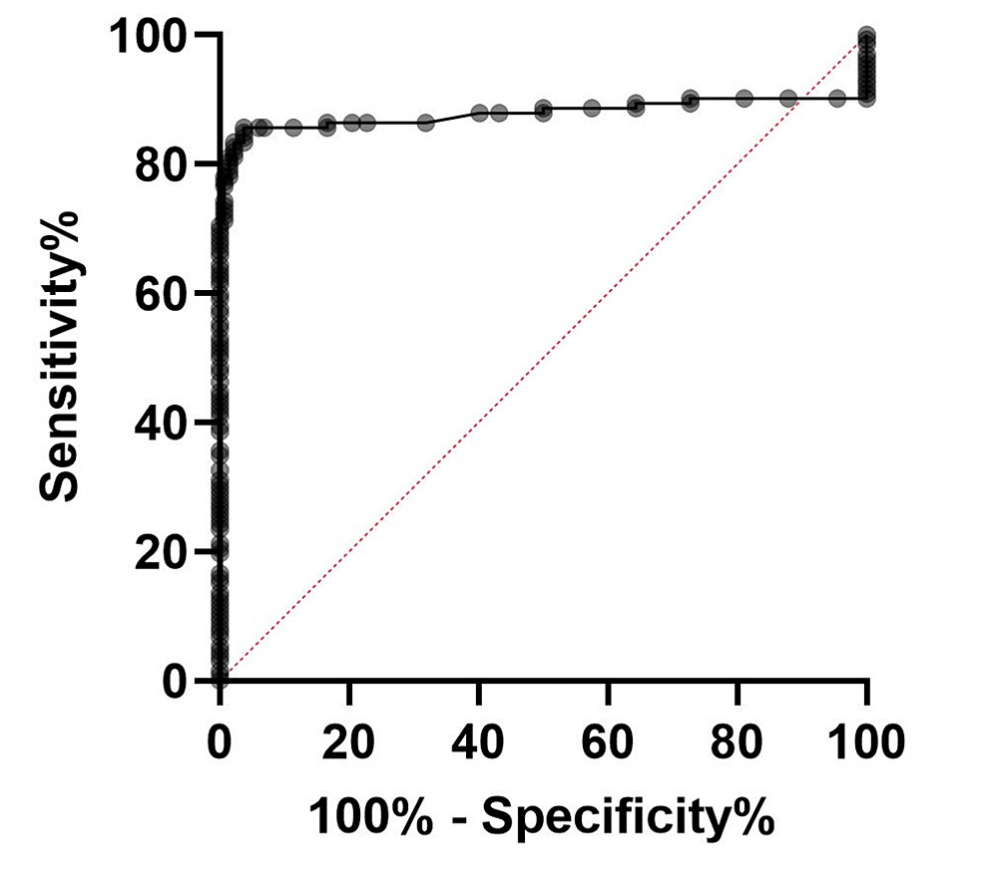
ROC curve of NLR vs HbA1C in the non-diabetic participants T2DM: type 2 diabetes mellitus; NLR: neutrophil-to-lymphocyte ratio; HbA1c: glycated hemoglobin

The ROC curve analysis to evaluate the diagnostic efficacy of NLR in comparison with HbA1c among T2DM patients showed an accuracy of 89.85% and superior sensitivity (85.46%) and specificity (94.23%), as shown in Figure 1, which is marginally better compared to ROC in non-diabetic participants (Figure 2). The ROC curve analysis to evaluate the diagnostic efficacy of NLR in comparison with HbA1c among the non-diabetic participants showed an accuracy of 87.82% and superior sensitivity (82.55%) and specificity (93.1%).

## Discussion

It is essential to manage T2DM patients to minimize long-term health complications. Despite neutrophil and lymphocyte counts being individually measured through the complete blood picture, the estimation of NLR has been suggested as a potential biomarker that can assess systemic inflammation and stress [7]. This is evident by the fact that neutrophils constitute the innate immune responses, and lymphocytes represent the adaptive/acquired immune responses. Among healthy individuals, the NLR ratio (1-2) is maintained. However, during infections and inflammatory or non-infectious conditions like cancers and other host disturbances, the NLR changes (>3 or <0.7). 

In recent times, especially during the coronavirus disease 2019 (COVID-19) pandemic, NLR has been used as a biomarker to manage COVID-19 patients. This was due to the evidence linking inflammatory status to abnormal NLR [8]. A very high NLR among COVID-19 patients suggested severe complications, hospitalizations, and mortality. 

The role of variations in the NLR has been explored in various non-infectious diseases like psoriasis, multiple sclerosis, neuromyelitis optica spectrum disorders, and breast cancer [9-12]. Similarly, the NLR ratio has been evaluated for its usefulness in managing infectious conditions like COVID-19, pneumonia, pertussis, diabetic foot ulcers/wounds, and urinary tract infections, especially among diabetic persons [13,14].

Additionally, T2DM is a known risk factor for infections and non-infectious conditions like invasive fungal diseases and chronic kidney and cardiovascular diseases [15-17].

T2DM is generally diagnosed and managed by biomarkers like the analysis of fasting blood glucose and HbA1C activity. However, there is emerging evidence of the utility of novel biomarkers like C-peptide and others like serum 25-hydroxyvitamin D (25(OH)D) in the diagnosis and management of T2DM [18,19]. 

T2DM predisposes people to complications like kidney diseases, cardiovascular complications, and other infectious and non-infectious outcomes. The utility of traditional biomarkers like estimation of blood glucose and HbA1c to manage T2DM appears to be limited. This is evident from the reports that suggest the potential application of NLR to manage T2DM and predict long-term complications like stroke and kidney and cardiovascular diseases, among others [20,21].

Only a few studies are available that have examined the role of NLR in managing T2DM patients. A previous study from Pakistan showed that an increased NLR correlated with abnormal glycaemic control, as evidenced by elevated HbA1c activity [22]. A recent systematic review and meta-analysis (SRMA) study by Adane et al. confirmed the role of NLR in predicting glycemic control among T2DM patients [23]. A study from China that included T2DM patients has positively evaluated the efficacy of NLR in predicting the prognosis of diabetic foot [24].

Study limitations

This hospital-based study included patients attending a tertiary care teaching hospital. The study included T2DM patients and healthy non-diabetic controls. The study results did not include variations in the NLR based on the co-morbidities present among the diabetic population. Therefore, NLR variations among T2DM patients were not attributed to a particular complication. Additionally, this study's results could have been influenced by the medication taken by the diabetic persons. Further studies in this regard are warranted to improve the understanding of the role played by NLR in the management of T2DM patients, especially with co-morbidities, including both infectious and non-infectious conditions.

## Conclusions

Despite the availability of traditional markers like HbA1c and blood glucose to diagnose and manage T2DM patients, they may be inappropriate to assess the overall health and predict long-term complications of T2DM. According to the results of the current study, there was a significant correlation between HbA1c and NLR among T2DM patients, unlike in non-diabetic persons. Moreover, the NLR significantly varied between T2DM patients and non-diabetic persons. The results of the current study support the role played by the NLR to manage T2DM patients. Alternatively, a more comprehensive evaluation of T2DM patients is possible with the estimation of NLR. Unlike the traditional biomarkers, NLR could be used to predict potential complications among T2DM patients.

## References

[1] (2024). World Health Organization: Diabetes. https://www.who.int/health-topics/diabetes?gclid=CjwKCAiAs6-sBhBmEiwA1Nl8s36O5sczZlvvtlPI_cYqRVGvMDV9SaCo5WFts7IbVj3wvm0jrwmZghoCkmcQAvD_BwE#tab=tab_1.

[2] (2024). Diabetes classification table. https://dtc.ucsf.edu/types-of-diabetes/diabetes-classification-table/.

[3] Bellou V, Belbasis L, Tzoulaki I, Evangelou E (2018). Risk factors for type 2 diabetes mellitus: an exposure-wide umbrella review of meta-analyses. PLoS One.

[4] Tummalacharla SC, Pavuluri P, Maram SR, Vadakedath S, Kondu D, Karpay S, Kandi V (2022). Serum activities of ferritin among controlled and uncontrolled type 2 diabetes mellitus patients. Cureus.

[5] Duman TT, Aktas G, Atak BM, Kocak MZ, Erkus E, Savli H (2019). Neutrophil to lymphocyte ratio as an indicative of diabetic control level in type 2 diabetes mellitus. Afr Health Sci.

[6] Butler AE, English E, Kilpatrick ES (2021). Diagnosing type 2 diabetes using hemoglobin A1c: a systematic review and meta-analysis of the diagnostic cutpoint based on microvascular complications. Acta Diabetol.

[7] Zahorec R (2021). Neutrophil-to-lymphocyte ratio, past, present and future perspectives. Bratisl Lek Listy.

[8] Buonacera A, Stancanelli B, Colaci M, Malatino L (2022). Neutrophil to lymphocyte ratio: an emerging marker of the relationships between the immune system and diseases. Int J Mol Sci.

[9] Hong J, Lian N, Li M (2023). Association between the neutrophil-to-lymphocyte ratio and psoriasis: a cross-sectional study of the National Health and Nutrition Examination Survey 2011-2014. BMJ Open.

[10] Zhou Q, Jia R, Dang J (2022). Correlation between the neutrophil-to-lymphocyte ratio and multiple sclerosis: recent understanding and potential application perspectives. Neurol Res Int.

[11] Fang X, Sun S, Yang T, Liu X (2023). Predictive role of blood-based indicators in neuromyelitis optica spectrum disorders. Front Neurosci.

[12] Arora R, Alam F, Zaka-Ur-Rab A, Maheshwari V, Alam K, Hasan M (2023). Peripheral neutrophil to lymphocyte ratio (NLR), a cogent clinical adjunct for Ki-67 in breast cancer. J Egypt Natl Canc Inst.

[13] Russell CD, Parajuli A, Gale HJ (2019). The utility of peripheral blood leucocyte ratios as biomarkers in infectious diseases: a systematic review and meta-analysis. J Infect.

[14] Făgărășan I, Rusu A, Comșa H, Simu TD, Vulturar DM, Todea DA (2023). IL-6 and neutrophil/lymphocyte ratio as markers of ICU admittance in SARS-CoV-2 patients with diabetes. Int J Mol Sci.

[15] Steinbrink JM, Miceli MH (2021). Mucormycosis. Infect Dis Clin North Am.

[16] Lao M, Li C, Li J, Chen D, Ding M, Gong Y (2020). Opportunistic invasive fungal disease in patients with type 2 diabetes mellitus from southern China: clinical features and associated factors. J Diabetes Investig.

[17] Shubrook JH, Neumiller JJ, Wright E (2022). Management of chronic kidney disease in type 2 diabetes: screening, diagnosis and treatment goals, and recommendations. Postgrad Med.

[18] Maddaloni E, Bolli GB, Frier BM, Little RR, Leslie RD, Pozzilli P, Buzzetti R (2022). C-peptide determination in the diagnosis of type of diabetes and its management: a clinical perspective. Diabetes Obes Metab.

[19] Wan Z, Song L, Hu L, Lei X, Huang Y, Lv Y, Yu S (2021). The role of systemic inflammation in the association between serum 25-hydroxyvitamin D and type 2 diabetes mellitus. Clin Nutr.

[20] Chang C, Zhou J, Chou OH (2023). Predictive value of neutrophil-to-lymphocyte ratio for atrial fibrillation and stroke in type 2 diabetes mellitus: the Hong Kong diabetes study. Endocrinol Diabetes Metab.

[21] Ciray H, Aksoy AH, Ulu N, Cizmecioglu A, Gaipov A, Solak Y (2015). Nephropathy, but not angiographically proven retinopathy, is associated with neutrophil to lymphocyte ratio in patients with type 2 diabetes. Exp Clin Endocrinol Diabetes.

[22] Hussain M, Babar MZ, Akhtar L, Hussain MS (2017). Neutrophil lymphocyte ratio (NLR): a well assessment tool of glycemic control in type 2 diabetic patients. Pak J Med Sci.

[23] Adane T, Melku M, Worku YB, Fasil A, Aynalem M, Kelem A, Getawa S (2023). The association between neutrophil-to-lymphocyte ratio and glycemic control in type 2 diabetes mellitus: a systematic review and meta-analysis. J Diabetes Res.

[24] Xu S, Wang Y, Hu Z, Ma L, Zhang F, Liu P (2023). Effects of neutrophil-to-lymphocyte ratio, serum calcium, and serum albumin on prognosis in patients with diabetic foot. Int Wound J.

